# Predictors of early attrition and successful weight loss in patients attending an obesity management program

**DOI:** 10.1186/s40608-016-0098-0

**Published:** 2016-03-09

**Authors:** Dishay Jiandani, Sean Wharton, Michael A. Rotondi, Chris I. Ardern, Jennifer L. Kuk

**Affiliations:** School of Kinesiology and Health Science, York University, Toronto, Canada; The Wharton Weight Management Clinic, Toronto, Canada; Room 2002B, Sherman Health Science Research Centre, York University, 4700 Keele St., Toronto, ON M3J 1P3 Canada

**Keywords:** Weight loss, Drop out, Overweight, Obese, Intervention, Clinical

## Abstract

**Background:**

Our objective was to identify factors that are independently associated with early attrition and successful weight loss (WL) in an obesity-management program.

**Methods:**

Participants were 9,498 patients enrolled in treatment at the Wharton Weight Management Clinic for at least 6 months. Predictors of early attrition (<6 months) and successful WL (≥5 %) were analyzed using relative risk (RR) in men and women separately. Pearson’s correlation was used to determine the relationship between WL and treatment time Weight loss and attrition analysis was restricted to patients who had more than two visits (*n* = 5415).

**Results:**

Older individuals had lower early attrition (RR Range:0.74–0.92, *P* < 0.05) and greater WL success (RR Range:1.40–1.65, *P* < 0.05) than younger individuals. Males with hypertension and females with depression had greater early attrition (RR Range:1.09–1.20, *P* < 0.05) and lower WL success (RR Range:0.48–0.57, *P* < 0.05) than those without these health conditions. Males with lower education had greater early attrition (*RR* = 1.11[1.03–1.19]) than males with higher education, but did not differ in WL. Females who smoked had greater early attrition (*RR* = 1.06[1.01–1.11]) than females who did not smoke, but did not differ in WL. Ethnicity was not related to early attrition, however, females of Black and Other ethnicities had lower WL success compared to White females (RR Range:0.58–0.74, *P* < 0.05). After adjusting for treatment time, all above associations were no longer significant and treatment time remained as the only independent predictor of WL success (*P* < 0.0001).

**Conclusion:**

As WL is positively and independently related with treatment time, individuals at risk for early attrition may need alternative treatment options, in order to improve patient retention and improve WL success.

## Background

High attrition is a common problem in weight loss interventions with attrition rates ranging from 10 % to more than 80 % depending on the type of intervention [1, 2]. Current literature identifies several characteristics that may predict attrition in weight loss programs such as age, sex, education, ethnicity and smoking status [1, 3–6]. However, the majority of the existing weight loss intervention literature focuses on research populations recruited using strict inclusion and exclusion criteria along with extensive baseline testing [4, 7–9]. For example, some studies report having patients pay for treatment [10] or require completion of at least 6 months of primary care for inclusion [9]. Studies also report excluding patients if they were unable to attend all baseline assessment or treatment sessions, or had existing obesity co-morbidities such as type 2 diabetes or hypertension [4]. Thus, patients recruited in these studies may be more motivated to complete treatment and are likely healthier than the general population with obesity. Due to the strict inclusion and exclusion criteria in research studies, the findings may not be generalizable to patients referred to weight management programs without these extra requirements [1, 3, 6, 11]. Understanding factors that influence early attrition and weight loss success in overweight and obese individuals seeking medical treatment is important, as it may lead to the implementation of alternative strategies, which may improve retention and ultimately health- and weight-related comorbidities. Therefore, our study aimed to examine factors related with early attrition and weight loss success in patients seeking medical weight management.

## Methods

### Participants

The Wharton Weight Management Clinic (WMC) is a multidisciplinary weight management clinic with several locations across Ontario, Canada (Toronto, Burlington, Hamilton, Stoney Creek and Etobicoke). The WMC assesses and provides weight management treatment for patients with overweight, obesity or type 2 diabetes using principles outlined in the Canadian clinical practice guidelines for the treatment of obesity [12]. Patients are referred to the clinic by their family physician and all visits are available free of charge to patients as services are covered by the Ontario Health Insurance Plan (OHIP). The clinic provides patients with a broad range of services such as physician assessments, blood pressure monitoring, ECG readings and other diagnostic tests. Patients are treated by a multidisciplinary team of behavioral therapists, dietitians, nutritionists, physicians, and bariatric educators. Patients are requested to visit the clinic every 4-6 weeks and all patients consult with a bariatric educator and a physician during each visit. On their first visit, all patients are required to complete a questionnaire which inquires about their goals and medical, social, psychological, diet and weight history. In addition, patients are also provided with an educational session on nutrition and are provided with program expectations. Patients then consult with a bariatric educator and physician in order to help set realistic goals and determine appropriate combination of treatments and nutrition which compliment their lifestyle. Physicians also create a plan to help treat complications and comorbities associated with obesity. Bariatric educators meet with all patients and monitor their nutrition and adjust their exercise and meal plan based on their needs and goals. After establishing their goals in the first visit, patients are in regular contact with the physician and bariatric educator who provide the patient with individualized weight management strategies using a combination lifestyle interventions such as diet and exercise. In addition, patients who are eligible for bariatric surgery are referred and provided with pre and post-operative care. Patients are encouraged to attend the clinic at least one a month in order to allow for monitoring or adjusting of the program to improve weight management strategies. Patients also may use additional resources such as counselors or psychological services if they require, based on physician referral from the clinic. As obesity is a chronic condition, the program has no specific length and patients are able to attend the clinic for as long as they need. On a regular visit, patients meet on an individual basis, however, additional information sessions are carried out in a group setting with 6-8 patients by various health professionals (i.e. psychologists, dieticians, kinesiologists etc). Patients also have the option to attend additional group information sessions which provide more specific information regarding diet, exercise and other related topics on weight management and health. For example, patients may attend an information session on “Emotional eating”. Individualized meal plans are provided by the bariatric educators for all patients who have regular follow-ups with their bariatric educators and physician on an appointment basis. In addition, patients that attend regularly are constantly monitored in order to adjust the strategies to optimize weight management. Participants provided written informed consent for the use of their electronic medical data for research purposes and were informed that participation or lack of participation would not affect their medical treatment. The methods used in this study were approved by York University Ethics Review Board.

### Attrition and group allocation

Data was extracted from electronic medical records on Dec 5, 2014 and included a total of 9498 participants who were at least 18 years of age. Participants were excluded from the analysis if they had missing or implausible values for age (*n* = 85), weight (*n* = 6), or initial visit date (*n* = 1). Participants were also limited to those who had enrolled at least 6 months prior to the data extraction date to allow for the correct categorization of patient attrition and weight loss outcomes and included a final sample of 8196. For our independent variables, age was defined as young (18–49 years), middle aged (50–64 years) and old (65+ yrs). Ethnicity was divided into 4 distinct subgroups with patients identifying themselves as White, Asian (South & East Asian), Black or Other. Education was stratified by those with less than or equal to high school education and those with greater than or equal to college. For smoking status, patients were divided into smokers or non-smokers with those who were past smokers being combined with smokers due to sample size. Individuals with health outcomes were divided in 6 distinct categories (CVD, Depression, Hypertension, T2D, Fatty Liver & Cancer) based on their diagnosis and coded as ‘yes’ to having the condition or ‘no’ if they did not have the specific condition.

For baseline characteristics, participants were divided into five mutually exclusive groups (single visit, <6 months and <5 % WL, <6 months and ≥5 % WL, ≥6 months and <5 % WL and ≥6 months and ≥5 % WL) based on the amount of time they attended treatment and their overall weight loss. Early attrition was defined as leaving treatment before 6 months and being absent for more than 6 months since their last visit. We used six months as it is thought to be the minimum length for a habit to be formed and weight management has many lifestyle components that need to be adopted as life long habits in order for patients to be successful [13]. Successful weight loss was defined as achieving a total body weight loss of ≥5 % of their initial body weight and weight loss was calculated using their last visit to clinic since the data extraction. Weight loss was calculated using difference between the patients’ last visit as of Dec 5th, 2014 and their first visit to the clinic. As patients may have enrolled at different times throughout the study period, the length of treatment differs from patient to patient (Range: 3.10–19.90 month). Weight loss and attrition analyses were restricted to participants with more than 2 visits (*n* = 5415).

### Statistical methods

Continuous variables were reported as means and SD while categorical variables were presented as frequencies and prevalences. Differences in baseline characteristics between each of the five groups were examined using one-way ANOVA with Bonferroni post-hoc tests for continuous variables and *X*^2^ tests for categorical variables**.** Kaplan-Meier survival analysis was used to illustrate patient attrition over treatment time. Independent *t-tests* were used to identify differences in both the absolute and the rate of weight loss between groups (<6 months and ≥6 months). Pearson’s correlation was used to determine the relationship between weight loss and treatment time. A sex-stratified Poisson regression analysis with mutual adjustments for age category (young vs. middle & old) initial BMI, ethnicity (White vs. Asian, Black & Other), education (college vs. high school), smoking status (smokers vs. non-smokers) and certain health conditions (cardiovascular disease (CVD), hypertension, depression, type 2 diabetes (T2D), fatty liver and cancer) were used to determine their independent associations with early attrition and successful weight loss (≥5 %). A second model included the additional adjustment for treatment time. Results were considered significant at *P* < 0.05 and all analyses were performed using SAS 9.4.

## Results

### Baseline characteristics

The baseline characteristics of the study participants stratified by treatment time (single visit, <6 months or ≥6 months) and weight loss status (<5 % or ≥5 %) are reported in Table 1. Participants were generally young, White (85.0 %) and female (74.8 %). Within the entire sample, 2779 (34.0 %) patients discontinued after a single visit. Of the 3057 (37 %) patients enrolled in treatment for greater than 6 months, 1242 (40.6 %) participants achieved successful weight loss (≥5 %).

**Table 1:** Baseline characteristics of patients by treatment time and weight loss status

Participant characteristics	Single visit	<6 months		≥6 months	
	No weight Loss	WL < 5 %	WL ≥ 5 %	WL < 5 %	WL ≥ 5 %
*N* = 8196	2779	1990	370	1815	1242
Female, %	76.2^cde^	77.2^cde^	72.7^ab^	71.7^ab^	72.7^ab^
Age, years	45.9 (13.0)^bcde^	48.1 (12.9)^acde^	49.2 (12.6)^abde^	52.2 (12.7)^abce^	54.4 (12.5)^abcd^
Initial Weight, kg	112.6 (25.0)	110.6 (23.9)^e^	112.3 (24.6)	112.7 (27.0)	114.7 (25.5)^b^
Weight change, %	-	1.0 (2.3)^cde^	8.8 (7.6)^bde^	−0.23 (4.2)^bce^	12.8 (9.1)^bcd^
Initial BMI, kg/m^2^	40.3 (7.6)^e^	39.8 (7.2)^e^	39.9 (7.8)	40.5 (8.2)	41.2 (7.9)^ab^
Final BMI, kg/m^2^	-	39.4 (7.2)^cde^	36.7 (6.6)^bd^	40.6 (8.4)^bce^	36.6 (7.2)^bd^
< High school	40.3^bd^	36.4^a^	36.7	36.6^a^	39.4
*Ethnicity*					
White, %	83.8^ce^	85.0^ce^	89.3^abd^	82.7^ce^	89.8^abd^
Asian, %	3.4^e^	2.1^ce^	2.4^ad^	3.2^ce^	1.3^abd^
Black, %	2.5	2.9	2.8	3.2^e^	1.6^cd^
Other, %	10.2^ce^	10.0^ce^	5.5^abd^	10.9^ce^	7.3^acd^
Current smokers, %	20.5^bcde^	14.8^ade^	12.7^a^	10.6^ab^	10.1^ab^
Treatment time, mos	-	3.1 (1.0)^de^	3.6 (1.1)^de^	19.5 (15.9)^bc^	19.9 (15.2)^bc^
*Baseline comorbidities*					
CVD, %	5.5	3.8	3.4^d^	6.7^c^	4.2
Hypertension, %	32.7^c^	35.3^cd^	38.6^abd^	34.1^bc^	35.6
Depression, %	16.5^bcde^	9.2^ace^	3.4^abd^	7.6^ac^	4.5^ab^
Type 2 diabetes, %	25.8^bde^	21.2^acde^	26.1^bde^	29.1^abc^	30.8^abc^
Fatty liver, %	14.8^bcde^	24.9^ade^	25.0^ad^	20.6^abc^	22.7^a^
Cancer, %	4.8^de^	5.6^e^	3.4^d^	1.9^ac^	2.1^ab^

*WL* weight loss, *BMI* Body Mass Index, *CVD* Cardiovascular disease, *CVD* heart attack, heart failure, thrombosis, stroke, hyperlipidemia

Bonferroni correction used for multiple comparisons

Values are in mean (SD) or prevalence (%) where indicated

^a^significantly different from single visit (*P* < 0.05)

^b^significantly different from <6 months with WL <5 % (*P* < 0.05)

^c^significantly different from <6 months with WL ≥5 % (*P* < 0.05)

^d^significantly different from ≥ 6 months with WL <5 % (*P* < 0.05)

^e^significantly different from ≥ 6 months with WL ≥5 % (*P* < 0.05)

### Weight loss and treatment time

Weight loss was positively related to treatment time (*r* = 0.38, *P* < 0.0001) and greater visit frequency (*r =* 0.43, *P* < 0.0001). Those in treatment for less than 6 months had lower absolute weight loss when compared with those in treatment for more than 6 months in males (2.8 ± 2.3 kg versus 6.1 ± 10.9 kg, *P* < 0.0001) and females (2.1 ± 4.9 kg versus 4.4 ± 8.7 kg, *P* < 0.001). However, patients in treatment for less than 6 months had a greater rate of weight loss in males (0.90 ± 1.80 kg/month versus 0.45 ± 0.76 kg/month, *P* < 0.0001) and females (0.68 ± 1.73 kg/month versus 0.37 ± 0.64 kg/month, *P* < 0.0001) when compared with those in treatment greater than 6 months.

### Predictors of early attrition

**Fig. 1:**
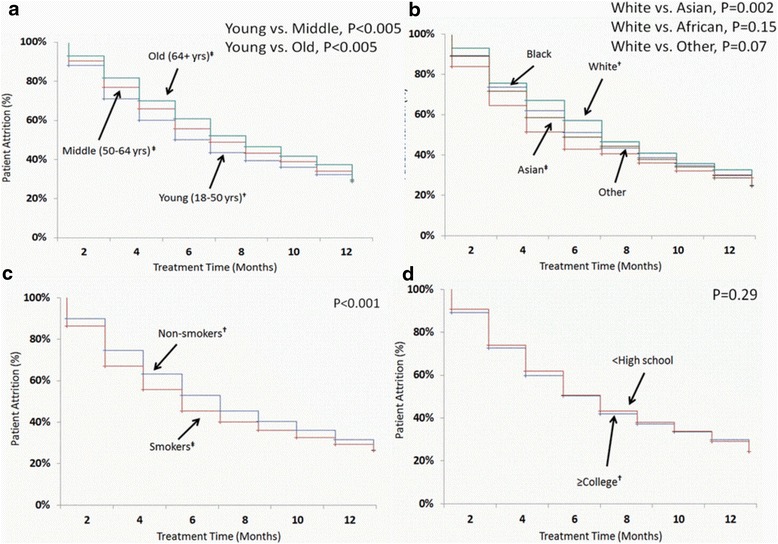
Kaplan-meier curve of patient attrition at WMC by: **a** Age, **b** Ethnicity, **c** Smoking Status and **d** Education. † indicates referent group. ‡ significantly different from referent group (*P* < 0.05)

Both middle-aged and older patients had lower early attrition rates (*P* < 0.005) than younger individuals (Fig. 1a). In comparison to White patients, Asians had higher early attrition rates (*P* = 0.002) but no differences were observed in early attrition rates of Black (*P* = 0.15) or Other ethnicities (*P* = 0.07, Fig. 1b). Patients who smoked had greater attrition rates when compared with patients who did not smoke (*P* < 0.001, Fig. 1c), whereas educational attainment was not related to early attrition (*P* = 0.29, Fig. 1d).

Table 2 reports the relative risk of early attrition by sex with mutual adjustments for age, initial BMI, ethnicity, education, smoking status and health conditions. Those who were middle-aged and older had lower early attrition (RR Range: 0.74–0.92, *P* < 0.05) when compared with younger individuals, regardless of sex. Having hypertension (RR Range: 1.16–1.20, *P* < 0.0001) was associated with greater attrition in both males and females. In females, having depression, fatty liver and a history of cancer predicted greater early attrition compared with females without these health conditions (RR Range: 1.09–1.27, *P* < 0.05), however, none of these conditions predicted early attrition in males (*P* > 0.05). Males with a lower education had greater early attrition (RR[95 % CI] = 1.11[1.03–1.19]) when compared with males with a higher education, but education did not predict early attrition in females. Ethnicity, smoking status, and the remaining health conditions were not related with early attrition in either males or females (*P* > 0.05).

**Table 2:** Mutually adjusted relative risk predicting likelihood of early attrition by sex

	Male		Female	
Participant characteristics	RRs (95 % CI)^a^	*P-value*	RRs (95 % CI)^a^	*P-value*
*Age*				
Young, 18-49 years	REF^b^	-	REF^b^	-
Middle, 50-64 years	0.92 (0.86–0.99)	*0.04*	0.91 (0.87–0.94)	*<0.0001*
Old, 65+ yrs	0.74 (0.66–0.84)	*<0.0001*	0.87 (0.83–0.93)	*<0.0001*
Initial BMI, kg/m^2^	1.06 (1.01–1.11)	*0.008*	1.03 (1.01–1.07)	*0.01*
Ethnicity				
White	REF^b^	-	REF^b^	-
Asian	1.08 (0.88–1.34)	*0.41*	1.10 (0.95–1.28)	*0.16*
Black	0.91 (0.74–1.12)	*0.40*	1.06 (0.94–1.18)	*0.32*
Other	0.94 (0.84–1.05)	*0.29*	0.97 (0.92–1.03)	*0.45*
*Education*				
> High school	REF^b^	-	REF^b^	-
≤ High school	1.11 (1.03–1.19)	*0.002*	1.00 (0.97–1.04)	*0.78*
*Smoking Status*				
Non-smokers	REF^b^	-	REF^b^	-
Current Smokers	0.96 (0.87–1.06)	*0.44*	1.06 (1.01–1.11)	*0.006*
*Health Outcomes*				
CVD	0.80 (0.56–1.14)	*0.23*	0.87 (0.71–1.06)	*0.17*
Hypertension	1.20 (1.10–1.30)	*<0.0001*	1.16 (1.10–1.22)	*<0.0001*
Depression	1.04 (0.71–1.50)	*0.85*	1.09 (1.01–1.19)	*0.03*
T2D	0.87 (0.74–1.03)	*0.11*	0.97 (0.91–1.05)	*0.59*
Fatty Liver	1.06 (0.91–1.23)	*0.46*	1.14 (1.06–1.22)	*0.0002*
Cancer	1.20 (0.95–1.51)	*0.11*	1.27 (1.18–1.35)	*<0.001*

*BMI* body mass index, *T2D* type 2 diabetes, *CVD* cardiovascular disease

^a^Adjusted for age, BMI, education, ethnicity and smoking status where appropriate

^b^REF indicates referent group

### Predictors of successful weight loss

Table 3 reports the relative risk of achieving successful weight loss (≥5 %) by sex with mutual adjustments for age, initial BMI, ethnicity, education, smoking status and health conditions. Similar to observations with attrition, older age also predicted greater likelihood of achieving successful weight loss (RR Range: 1.40–1.65, *P* < 0.05) when compared with younger individuals, in both males and females. In females, those who were Black (RR[95 % CI] = 0.58[0.37–0.94]) or belonged to ‘Other’ ethnicities (RR[95 % CI] = 0.66[0.57–0.94]) had a lower likelihood of successful WL compared with White individuals. Females with depression were also less likely to attain a 5 % weight loss (RR[95 % CI] = 0.48[0.27–0.85]) than females without depression, but no differences were observed for all other health conditions (*P* > 0.05). In males, only hypertension was associated with lower WL success (RR[95 % CI] = 0.57[0.40–0.81]). After adjusting for treatment time, there were no differences in WL success regardless of age, smoking status, ethnicity or health conditions (*P* > 0.05) in either males or females (*P* > 0.05).

**Table 3:** Mutually adjusted relative risk predicting likelihood of achieving successful weight loss by sex

	Male		Female	
Participant Characteristics	RRs (95 % CI)^a^	*P-Value*	RRs (95 % CI)^a^	*P-Value*
*Age*				
Young, 18-49 years	REF^b^	*-*	REF^b^	*-*
Middle, 50-64 years	1.40 (1.12–1.77)	*0.003*	1.42 (1.23–1.64)	*<0.0001*
Old, 65+ yrs	1.50 (1.14–1.98)	*0.004*	1.65 (1.39–1.96)	*<0.0001*
Initial BMI, kg/m^2^	0.93 (0.88–0.98)	*0.008*	0.95 (0.92–0.99)	*0.01*
Ethnicity				
White	REF^b^	*-*	REF^b^	*-*
Asian	0.74 (0.40–1.38)	*0.35*	0.74 (0.46–1.19)	*0.22*
Black	1.03 (0.57–1.88)	*0.91*	0.58 (0.37–0.94)	*0.03*
Other	0.78 (0.53–1.13)	*0.18*	0.74 (0.57–0.94)	*0.02*
*Education*				
> High school	REF^b^	*-*	REF^b^	*-*
≤ High school	0.89 (0.74–1.06)	*0.21*	1.05 (0.94–1.18)	*0.36*
*Smoking Status*				
Non-smokers	REF^b^	*-*	REF^b^	*-*
Smokers	0.89 (0.67–1.18)	*0.42*	0.82 (0.67–1.00)	*0.05*
*Health Outcomes*				
CVD	0.65 (0.30–1.45)	*0.30*	0.64 (0.35–1.14)	*0.13*
Hypertension	0.57 (0.40–0.81)	*0.001*	0.97 (0.80–1.18)	*0.80*
Depression	0.37 (0.06–2.45)	*0.30*	0.48 (0.27–0.85)	*0.01*
T2D	1.02 (0.78–1.36)	*0.85*	0.94 (0.76–1.16)	*0.57*
Fatty Liver	0.98 (0.62–1.53)	*0.92*	0.97 (0.72–1.32)	*0.86*
Cancer	0.72 (0.22–2.36)	*0.59*	0.49 (0.22–1.09)	*0.08*

*BMI* body mass index, *T2D* type 2 diabetes, *CVD* cardiovascular disease

^a^Adjusted for age, BMI, education, ethnicity and smoking status where appropriate

^b^REF indicates referent group

## Discussion

A majority of the existing research on attrition has been conducted in strictly controlled clinical trials, and may not be generalizable to the general population seeking weight management. In the general population seeking weight management, we observe that younger age and certain health conditions were associated with both early attrition and lower WL success in both sexes. In addition, lower educational attainment in males and smoking status in females predicted only early attrition whereas, females of ethnic minorities only had lower WL success. As none of the characteristics were associated with WL success after adjusting for treatment time, these results suggest that greater time in treatment may be beneficial for WL success.

We observe that the duration of treatment was an independent predictor of weight loss, which is in accordance with previous literature [13, 14]. Longer treatment times may be important as it may allow for continued support [13], and provide patients with a greater opportunity to practice the behaviors necessary for long term WL success [14], which may aid in reducing attrition and improve weight loss success. Conversely, greater weight loss may be the motivating factor leading to longer treatment length, however, we and others [13, 14] observe that those with longer treatment time had a lower rate of weight loss. Thus, as the rate of weight loss is lower, it is unlikely that the weight loss is the motivating factor for patients to remain in treatment. Given the low success of independent weight management after formal weight loss programs cease [13, 15], it may be important to reframe obesity as a chronic condition and provide chronic clinical care.

Our study reports that older age is associated with lower early attrition and greater weight loss success, which is in accordance with some studies [6, 9] but in contrast with others that report no influence of age on attrition [16–18]. The studies that did not observe associations between age and attrition had smaller age ranges consisting of mainly younger or middle-aged participants [16–18], as opposed to our study that also included younger, middle-aged and older adults. These age-related differences in attrition may be explained by several factors. Younger individuals may not be able to attend treatment as frequently as older individuals as they may have the extra burden of childcare [6], have less financial stability [6], may not be able to take time off work [6], or may be less motivated to improve their health [19]. In some settings, patients seeking medical treatment from weight management clinics may also be faced with additional barriers such as long wait times or distance to the clinic. As attrition and weight loss are related, the lower weight loss success in younger patients as compared to older individuals may also be due to their greater early attrition. Therefore, there may be a need to adopt specific strategies that cater to younger individuals, in order to improve treatment attendance and weight loss success.

Previous literature on attrition and weight loss often excludes participants who have existing obesity-related health conditions [16, 20, 21] such as T2D [22], depression, or a history of cancer [23] while others make no mention of participant health conditions [7, 17, 24]. In our study, having certain baseline comorbidities such as depression or hypertension is associated with both greater early attrition and lower WL success depending on sex. Our findings are consistent with previous studies [19, 22, 25], but contradict several others that do not observe comorbidities to be associated with differential attrition or weight loss [18, 26]. For example, having depression may interfere with weight management as it is often associated with symptoms such as lethargy and lack of motivation [25], or uncontrolled eating and substance abuse [25], which may make weight loss or attendance more difficult. The remaining health conditions such as hypertension or cancer may be related to attrition as they may reduce quality of life [27] which may in turn increase attrition and reduce weight loss success. However, the relationship between hypertension, cancer and their effect on WL and early attrition is less clear and warrants future investigation. Other factors such as the use of medications are also important to consider as they can be associated with weight gain [28]. Therefore, participants may require more flexible treatment options and a more tailored approach in order to improve WL success and reduce early attrition depending on their comorbid conditions.

Lower educational attainment was related to greater early attrition in only males, and was not related with weight loss success in either sex. Our findings are consistent with some previous research studies [21, 29] which report an association between education and attrition, but differ from others which report no relationship between education and attrition [10, 16]. Education may be related to greater early attrition as education is often a marker of SES [21]. SES may be associated with lower weight loss success in patients of ethnic minorities, however, income was poorly completed in our study because of which we could not include SES information in our study. As the clinic is publically funded, patients often questioned the importance of completing questions related to income as the program is offered at no cost to the patients. As a result, our study used education as it is often related to health literacy and income [1, 21, 30]. Therefore, patients with lower education or SES may have greater attrition due to inflexible work hours [21, 29], transportation or parking costs, or reliance on public transportation which may make it more difficult to attend treatment. Although education was associated with greater early attrition, education level did not influence weight loss success in our study. The program at WMC is purposefully designed so that it will be understandable and accessible to those with more modest English language facility and health literacy. Patients are also in regular contact with their physician, which is important in building a trusting and collaborative physician-patient relationship which has been reported to help overcome obstacles to weight loss [24]. Thus, while low educational attainment does not appear to limit weight loss success, it may still be associated with greater early attrition in males. Additional accommodations may therefore be necessary to reduce early attrition in patients with lower education.

With regard to smoking status and attrition, the current literature has reported mixed findings [9, 10, 18, 19, 21, 25]. Two studies [9, 19] demonstrated no relationship between smoking status and attrition in patients in a randomized weight loss trial [19] and a clinical weight management program [9]. However, these studies frequently contacted patients who missed program visits [9] or had a long run-in period prior to study inclusion [19], which may have improved treatment compliance and made it harder to discern differences in attrition by smoking status. In our study, we observe greater early attrition in patients who smoke. Patients who smoke might have been discouraged from attending weight management treatment after physician consultations regarding the negative effects of smoking. Additionally, as smoking cessation is often associated with weight gain [31], it may be particularly discouraging for patients seeking weight management treatment. However, although smoking status was related with greater attrition, it was not related with differences in weight loss in our study. Taken together, these findings suggest that these individuals may not be attempting to stop smoking or that the treatment provided may be adequate in combatting post-cessation weight gain. As early attrition continues to be a challenge, particular attention should be focused on reducing early attrition in patients who smoke.

Currently, relatively few studies have been adequately powered to examine differences in attrition by ethnicity as most studies have often reported small samples of ethnic minorities [*n* = 57 to 78] [6, 19, 22]. For example, the only study to report greater attrition in Black patients compared to White individuals attending a clinic based weight loss program had the largest sample of ethnic minority patients [*n* = 78] [6]. In contrast, two studies [19, 22] reported no association between ethnicity and attrition but had comparable sample sizes of Black [*n* = 61] [22] and Other [*n* = 76] [19] ethnic patients to the above study [6]. Although we had a significantly greater number of non-White patients [*n* = 1229] from a more diverse ethnic background as compared to the mentioned studies, we also did not observe differences in attrition between patients who were White, Black, Asian or Other ethnic background. This may be due to the ethnic diversity of the staff at WMC, which may make the patients feel more comfortable and remain in the program longer. Although ethnicity did not influence attrition, females of ethnic minorities had lower weight loss success compared to White females. Our findings are similar to the findings of Fabricatore et al. [19] who reported lower WL success in Black individuals when compared to White individuals. Individuals of ethnic minorities may have lower WL success, as the majority of the staff at the clinic are trained in providing dietary advice using predominantly North American foods. This may make the WMC dietary intervention less effective for those who normally consume their own ethnic foods. In addition, differences in weight management outcomes may also be due to physiological differences such as resting metabolic rate [19] or language barriers [32]. Given the differences in weight loss success amongst ethnic groups, it may be important to provide strategies tailored to these ethnic differences as it may improve WL success, particularly as many of these ethnicities are reported to be more likely to have obesity related comorbidities at lower levels of obesity.

Therefore, we observe that patients that are in treatment for longer than 6 months tend to see greater weight loss compared to those in treatment for less than 6 months. As weight loss is quite variable in our study ranging from 3 months to 20 months, the differences in the length of treatment between individual patients may account for and impact the overall weight loss reported in our study. Our results demonstrate that both weight loss and treatment time are related, as those who have longer treatment time observe larger weight loss success [13, 15]. Although majority of the patients discontinue treatment before 6 months, WMC makes an attempt to reduce attrition by encouraging more frequent visits (once every 4-6 weeks) in order to continue assisting with weight loss with the eventual goal of weight maintenance. In addition, more frequent visits have also been related to less abandonment of treatment and greater weight maintenance. As previous research [33] indicates a diminishing of weight loss around 6 months, our program tries to focus towards behaviours of weight maintenance. Currently, our program mirrors the real life obstacles faced by patients who are in weight management programs and thus we suggest that patients attend clinic regularly in order to improve treatment success, maximize their weight loss and practice behaviours necessary for weight maintenance.

Our analysis has several strengths and limitations. To our knowledge, this is the first study with such a large ethnically diverse clinical sample. This large sample allowed us to stratify our findings by sex as opposed to statistical adjustment, which is important given the clear sex differences that are commonly observed in health and obesity research. Our sample also consisted of patients that are often excluded from clinical trials. Despite these strengths, our study also has several limitations. Due to the observational study design, our findings cannot imply causation. Furthermore, there are many other factors that we did not assess which may have an effect on attrition and weight loss, such as eating behaviors (weight cycling, binge eating), logistics (travel distance, income, environment), personality and physical health.

## Conclusion

In conclusion, our study determined that in a physician-referred weight management population, longer treatment time is the only independent predictor of weight loss. We also observed greater early attrition and lower WL success in younger individuals and those with certain health conditions, regardless of sex. Other factors such as education and smoking status were only related with early attrition, whereas individuals of ethnic minorities had no differences in attrition but were still less likely to lose weight. As early attrition and WL are generally related, using a range of strategies to minimize early attrition in groups at risk may improve program adherence and increase WL success.
